# Differentiation of true transient ischemic attack versus transient ischemic attack mimics

**Published:** 2014-07-04

**Authors:** Ali Noureddine, Kavian Ghandehari, Mohammad Taghi Shakeri

**Affiliations:** 1 Department of Neurology, School of Medicine, Mashhad University of Medical Sciences, Mashhad, Iran; 2 Department of Neurology, Neuro cognitive Research Center, School of Medicine, Mashhad University of Medical Sciences, Mashhad, Iran; 3 Department of Social Medicine, School of Medicine, Mashhad University of Medical Sciences, Mashhad, Iran

**Keywords:** Transient Ischemic Attack, Clinic, Symptom, Mimics

## Abstract

**Background: **Previous literatures have shown a transient ischemic attack (TIA) mimic rate of 9-31%. We aimed to ascertain the proportion of stroke mimics amongst suspected TIA patients.

**Methods:** A prospective observational study was performed in Ghaem Hospital, Mashhad, Iran during 2012-2013. Consecutive TIA patients were identified in a stroke center. The initial diagnosis of TIA was made by the resident of neurology and final diagnosis of true TIA versus TIA mimics was made after 3 months follow-up by stroke subspecialist.

**Results: **A total of 310 patients were assessed during a 3-month period of which 182 (58.7%) subjects were male and 128 (41.3%) were female. Ten percent of the patients was categorized as a TIA mimic. The presence of hypertension, aphasia, duration of symptoms, and increased age was the strongest predictor of a true TIA. Migraine was the most common etiology of stroke mimic in our study.

**Conclusion: **It seems that many signs and symptoms have low diagnostic usefulness for discrimination of true TIA from non-cerebrovascular events and predictive usefulness of any sign or symptom should be interpreted by a stroke neurologist.

## Introduction

The traditional diagnosis of transient ischemic attack (TIA) is clinical and not based on any specific diagnostic test. TIA is an impressive warning of stroke, and its recognition provides the opportunity for therapeutic intervention. TIA usually occurs when a physician is not available to examine the patient.^1^ By definition, all patients with a TIA or stroke will have had focal neurological symptoms, which are those that arise from a disturbance in an identifiable and localized area of the brain.^1^ The symptoms duration of 24 h was chosen to distinguish TIA from stroke. This time limit has been widely accepted. The patient must have no abnormal neurologic symptoms between attacks, unless there has been previous infarction.^1^ Patients with a TIA have a high-risk of stroke within the first 3 months, ranging from 9% up to 20% at 90 days.^1^^,^^2^ High-risk of stroke in the first few days shows the need for urgent evaluation and treatment of patients with TIA.^1^^,^^3^^,^^4^ However, there are patients with transient focal neurological symptoms, which are not attributable to a focal cerebral ischemia.^5^ Such conditions may imitate a TIA and can therefore be labeled TIA mimics, in analogy to conditions that copy a stroke and have been labeled stroke mimics.^6^^-^^8^ The rate of TIA mimics ranges from 10% to 48.5% depending on the setting.^5^^,^^8^^-^^10^ Diagnostic procedures and therapeutic measures differ between patients with true TIA and patients with TIA mimics. Therefore, it would be useful to identify clinical features, which aid in discriminating between the two categories. We hypothesized that some differences in clinical characteristics of TIA versus TIA mimics could be useful for differentiation of these entities. We compared these characteristics of patients with true TIA and patients with TIA mimics presenting to a stroke center.

## Materials and Methods

Consecutive patients with initial diagnosis of TIA referred to the stroke center of Ghaem Hospital, Mashhad, Iran entered the study during 2012-2013. All of the patients had a clinical neurological exam on admission by a resident of neurology and TIA was detected based on the standard definition as an acute loss (i.e., within seconds) of focal cerebral or ocular function with symptoms lasting up to 24 h and of presumed ischemic origin.^1^^,^^4^ Although some stroke leaders proposed a new definition of TIA as focal ischemic neurological deficit lasting <1 h without new ischemic changes in magnetic resonance imaging (MRI) (diffusion weighted imaging and fluid attenuated inversion recovery sequences) this new definition did not gain worldwide agreement and we have not used this definition in our research.^1^ All of our TIA patients had a routine brain computed tomography and MRI was performed in selected patients. All of the presumed TIA cases had a follow-up visit after 1 week and 3 months period by a stroke neurologist. All subjects with a primary non-cerebrovascular disorder were classified as having TIA mimics. Stroke neurologist assessed case report forms and clinical data, and reclassified the patients as true TIA or TIA mimics. The diagnosis of TIA or TIA mimics was based on clinical criteria alone.^1^^,^^5^^,^^7^ Detection of TIA mimics, for example, conversion disorders was made by stroke neurologist based on the routine knowledge of cerebrovascular disease^1^ and American Stroke Association Guidelines 2013 (see: http://stroke.ahajournals.org). All of the studied patients had blood chemistry, Doppler ultrasound of neck arteries and an electrocardiogram. Patients who refused performing the necessary diagnostic investigations were excluded. Patients who did not have follow-up visits at 1 week and after 3 months period and cases who did not sign informed consent were also excluded. Data were collected on an identical case report form. The following variables were assessed: demographic variables (age and sex), details of clinical symptoms, symptoms duration, and recurrence of symptoms in 1^st^ week and at 3 months of assessment, blood pressure, diabetes mellitus, hyperlipidemia, smoking, and carotid stenosis. We collected information on six clinical symptoms: unilateral paresis, unilateral plegia, unilateral sensory loss or paresthesia, aphasia, dysarthria, and amaurosis fugax. The Ethics Committee of Mashhad University of Medical Sciences approved the study protocol in a proposal coded 2630.

***Statistical analysis***

Data were entered in the Statistical Package for Social Sciences (SPSS) for Windows 16.0 (Chicago, IL, USA) software package. We used descriptive statistics with absolute and relative frequencies for categorical variables and median for continuous variables to study differences in the distribution of patient characteristics at baseline between patients with true TIA and TIA mimics. The P values calculated using Fisher’s exact test and the Mann-Whitney test, respectively.

## Results

A total of 310 patients with initial diagnosis of TIA were reviewed of which 182 (58.7%) subjects were male, and 128 (41.3%) subjects were female. Patient’s age ranged 24-92 years with a mean age of 63.39 ± 13.08. Thirty patients (9.67%) were classified as a stroke mimic. Mean age of the patients having TIA mimic and true TIA was 50.40 ± 15.52 and 64.78 ± 12.02 years, respectively (P = 0. 001). There was no significant difference between recurrent episode of the TIA in the 1^st^ week of assessment and type of TIA (P = 0.398). Table 1 represents clinical characteristics of our two groups of initially detected TIA patients.

**Table 1:** Characteristics of the study population and relation of presenting signs or symptoms with true transient ischemic attack and transient ischemic attack mimic

**Item**	**TIA mimic**	**True TIA**	**P**
Male, n (%)	18 (60.0)	164 (58.6)	0.8800
Hypertensive, n (%)	13 (43.3)	190 (67.9)	0.0007
Hemiparesis, n (%)	19 (63.3)	188 (67.1)	0.6740
Hemiplegia, n (%)	4 (13.3)	38 (13.6)	0.9710
Aphasia, n (%)	6 (20.0)	6 (2.1)	0.0001
Dysarthria, n (%)	8 (26.7)	132 (47.1)	0.0320
Paresthesia, n (%)	9 (30.0)	78 (27.9)	0.8040
Amaurosis fugax, n (%)	30 (100.0)	272 (97.1)	0.3480
Diabetes mellitus, n (%)	5 (16.7)	70 (25.0)	0.3110
Hyperlipidemia, n (%)	6 (20.0)	87 (31.7)	0.2090
Smoking, n (%)	3 (10.0)	46 (16.4)	0.3590
Carotid stenosis, n (%)	0 (0.0)	15 (5.4)	0.1940
Symptom repetition	2.26 ± 3.07	1.80 ± 2.79	0.3980

The presence of hypertension, aphasia, dysarthria, duration of symptoms, and increased age was the strongest predictors of a true TIA. There was a significant difference between symptom duration in true TIA and TIA mimic groups (P = 0.004). Table 2 differentiates two groups of initial TIA patients based on the symptom duration categories. True TIA and TIA mimic groups were more preponderant for symptoms duration 10-60 min and more than 60 min, respectively.

A total of 26 of 30 (86.6%) TIA mimics in our study were neurological disorders. Sixty-three percent of patients with TIA mimics had migraine with aura, and the aura was confused with TIA manifestations. Visual, motor, sensory, and speech auras constituted 90%, 40%, 72%, and 12% of aura subtypes, respectively, which were often mixed with each other. The causes of stroke mimic are detailed in table 3.

**Table 2:** Distribution of symptoms duration in patients with true transient ischemic attack and transient ischemic attack mimic

**Duration category**	**TIA mimic**	**True TIA**
Less than 10 min, n (%)	9 (30.0)	74 (26.4)
Between 10 and 60 min, n (%)	2 (6.7)	99 (35.4)
More than 60 min, n (%)	19 (63.3)	107 (38.2)

TIA: Transient ischemic attack

**Table 3:** Frequency rate of causes of stroke mimics (n = 30) in our patients

**Final diagnosis**	**Total number**	**Percentage**
Migraine	19	63
Seizure	5	16
Conversion disorder	4	13
Primary brain tumor	1	3
Metastatic brain tumor	1	3
Total	30	100

## Discussion

Neuroimaging and paraclinical investigations can improve the diagnosis of TIA.^1^^,^^11^ However, bedside clinical judgment remains main stone in differentiation of true versus false TIAs.^1^^,^^11^ Our prospective study showed the following main results: (1) About 1 out of 10 patients with initial diagnosis of TIA made by a neurologist had a TIA mimic rather than a true TIA. (2) Weakness as a TIA symptom is a known predictor of consecutive stroke and represents an important element in clinical scores used to predict stroke risk in patients with true TIA,^12^ but in our study hemiparesis or hemiplegia was not a predictor of a true TIA. (3) Some of the predictive capability of the ABCD2 or ABCD score may be clarified by the fact that patients with low scores may in fact have a TIA mimic rather than a true TIA. (4) Blood pressure values differ between patients with TIA mimics versus true TIA patients. This shows that true TIA patients have significantly higher blood pressure values than patients with TIA mimics.^11^^,^^13^ Speech and language disturbances was significantly more frequent in patients with true TIA than TIA mimics patients in our study. This finding is considered in acute stroke assessment tools; for example, speech is a component of the face arm speech test, the Cincinnati Pre-hospital Stroke Scale^10^^,^^14^ and the recognition of stroke in the emergency room scale.^9^^,^^15^^-^^17^ Our study demonstrated that differentiation between disorders mimicking TIA and true TIAs is also relevant with regard to the short-term risk of vascular events, which has been evaluated in our center.^18^^,^^19^ Differences in management and source allocation between patients with true TIA and TIA mimics are therefore justified. Relatively small number of patients with TIA mimics forced us to restrict the numerous possible clinical symptoms. In general, our data will be helpful to distinguish between true TIA and TIA mimics. The value of some of TIA signs or symptoms needs to be evaluated in more detail. The frequency of TIA-mimicking diagnoses identified in our study subjects was similar to Purroy et al. report.^5^ Many mimics are seen rarely, such as transient global amnesia, demyelinating disorder, primary or metastatic brain tumors, spinal cord lesions, and so on.^20^^-^^23^ Because cerebrovascular accident is a clinical diagnosis, our data reinforce the need for stroke physicians to be responsible in the evaluation of patients with brain attack or TIA.^24^

## Conclusion

Among patients with suspected TIA in our study, 1 out of 10 patients had a TIA mimic. Patients with TIA mimics were approximately 14 years younger than those with true TIA. Language or speech abnormalities, duration of the symptoms, high blood pressure values, suggested the diagnosis of true TIA, while paresis, sensory symptoms, amaurosis fugax, hyperlipidemia, and diabetes mellitus were not shown to distinguish between the two entities.

## Notes

**How to cite this article: **Noureddine A, Ghandehari K, Shakeri MT. Differentiation of true transient ischemic attack versus transient ischemic attack mimics. Iran J Neurol 2014; 13(3): 127-30.
